# CCR2 Defines a Distinct Population of NK Cells and Mediates Their Migration during Influenza Virus Infection in Mice

**DOI:** 10.1371/journal.pone.0052027

**Published:** 2012-12-13

**Authors:** Mary J. G. van Helden, Dietmar M. W. Zaiss, Alice J. A. M. Sijts

**Affiliations:** Department of Infectious Diseases and Immunology, Faculty of Veterinary Medicine, University of Utrecht, Utrecht, The Netherlands; McMaster University, Canada

## Abstract

Natural killer (NK) cells are innate lymphocytes that play an important role in control of viral infections. We recently showed that intranasal infection of mice with influenza virus induced the accumulation of NK cells in the airways. NK cells however did not proliferate in the airways or in the draining lymph node, but in the bone marrow mainly. As also monocyte-precursors undergo vigorous proliferation in the bone marrow (BM) during infections and then egress CCR2-dependently, we decided to determine the role of CCR2 in NK cell migration during intranasal influenza virus infection. We show that a unique population of NK cells in the BM expressed CCR2 and that monocyte chemotactic protein-1 (MCP-1), one of the CCR2 ligands, was produced in the airways of influenza virus infected mice. Analysis of BM chimeric mice reconstituted with a mix of *wild-type* (*wt*) and CCR2-deficient BM cells showed that upon influenza virus infection, a significantly lower proportion of CCR2-deficient than *wt* NK cells was recovered from the bronchoalveolar lavage (BAL). Taken together, our data demonstrate that during influenza virus infection a proportion of NK cells migrate in a CCR2-dependent fashion.

## Introduction

Natural killer (NK) cells provide early control of viral infections by killing infected cells and by cytokine production [1]. NK cells respond to upregulation of activating ligands on target cells, which in case of viral infections can be of cellular origin [2], or pathogen-encoded [3]. NK cells also sense downregulation of MHC class I receptors, which are ligands for inhibitory receptors on the NK cell surface, and in this way help to eradicate viruses that escape CD8 T cell mediated-immunity [4].

The bone marrow (BM) has been viewed traditionally as the main site for NK cell development [5], although recent papers have shown that development can also occur in the thymus [6], lymph nodes [7] and the liver [8], [9]. The differentiation of NK cell progenitors into fully mature NK cells can be divided into separate stages that are marked by the up- or downregulation of distinct receptors [5], [10] like CD27 and CD11b [10]–[12], KLRG1 [13] and CXCR3 [14]. Upon maturation, NK cells start to express sphingosine 1-phosphate receptor 5 (S1P_5_), and NK cells were shown to accumulate in the BM of S1P_5_-deficient mice, indicating that this receptor is responsible for egress from the BM, or it may prevent exit of NK cells from blood vessels [15]. NK cell were not completely absent in the periphery of S1P_5_-deficient mice, indicating that other chemokine receptors contribute to egress from the BM [15]. The group of Santoni showed that CXCR4 expression is progressively decreased during maturation of NK cells and that administration of the CXCR4 antagonist AMD-3100 induced NK cell egress from the BM [16]. They proposed a model in which CXCR4 mediates retention of immature NK cells in the BM, and the combination of CXCR4 downregulation and S1P_5_ upregulation during maturation induces egress of mature NK cells from the BM [17].

Although NK cell are known to express a large variety of chemokine receptors [18]–[23], the ‘chemokine codes’ leading to NK cell accumulation at sites of infection still remain largely unknown [24]. The chemokine receptors involved in NK cell migration to the site of infection have been assessed for a few pathogens, and from these studies it appeared that CCR2, CCR5, CXCR3 and CX3CR1 play a key role in NK cells migration during infection [25]. Interestingly, a cascade of innate chemokines leading to NK cell recruitment during MCMV infection has been unraveled, in which type I interferon (IFN)-induced monocyte chemotactic protein-1 (MCP-1) production by resident F4/80^+^, probably kupfer cells, induces the influx of macrophage inflammatory protein 1 alpha (MIP-1α)-producing macrophages. These macrophages, in turn produce MIP-1α, which induces NK cell recruitment [26]–[28]. The direct role of MCP-1 on NK cells, however, was not determined in these studies.

We have shown recently that NK cells accumulated in the airways of influenza virus infected mice, but failed to proliferate there. Instead, we found NK cell expansion to occur in the BM [29]. This is similar to the proliferative response of monocyte precursors in the BM of *Listeria monocytogenes* infected mice [30], which then emigrate CCR2 dependent into the bloodstream [31]. During Listeria infection, chemokine receptor-mediated signalling is required for egress from the BM only, as CCR2^−/−^ or pertussin toxin-treated monocytes injected into the bloodstream of *L. monocytogenes*-injected mice readily migrate to the site of infection [31], [32]. This finding can be extended to influenza virus infection, where TNF-α/inducible nitric oxide synthase (iNOS)-producing DCs (tipDCs) failed to accumulate in the airways of influenza virus-infected CCR2^−/−^ mice [33].

As NK cells and monocytes share their ability to proliferate in the BM during infection, we here aimed to identify whether CCR2 plays a key role in migration of NK cells also during influenza virus infection.

## Materials and Methods

### Mice and Infection

C57BL/6 mice were purchased from Charles River and CCR2^−/−^ on a C57BL/6 background from Jackson. C57BL/6.SJL (CD45.1), CD45.1.2 (F1 of C57BL/6×C57BL/6.SJL) and CCR2^−/−^ mice were bred in house under standard conditions. Mice were used between 7–17 weeks of age. Intranasal (*i.n.*) influenza virus infection (A/HK/x31; H3N2) was performed with 10^5^ 50% egg infective dose in 30 ul PBS as described [34] under light isofluorane anesthesia.

### Ethics Statement

Animal experiments were performed in agreement with the Dutch Animal Experimentation Act and EU directives 86/609/CEE and 2010/63/EU related to the protection of vertebrate animals used for experimental or other scientific purposes. All experimental protocols were approved by the Committee on Animal Experiments of the University of Utrecht and performed in the Central Laboratory Animal Research Facility of the University of Utrecht, which has AAALAC (Association for Assessment and Accreditation of Laboratory Animal Care) accreditation and all efforts were made to minimize animal suffering.

### Sample Collection and Tissue Preparation

Mice were sacrificed by intraperitoneal (*i.p*.) injection of sodium pentobarbital and lymphocytes were obtained from the spleens, BM and lungs or bronchoalveloar lavage (BAL). For BAL, the thorax was opened and the trachea exposed. A small aperture was made with scissors, a small tube (0.965 mm polyethylene tubing, Becton Dickinson) attached to a syringe was inserted and fastened with a knot using suture thread. BAL was collected by three times lavage with 1 ml PBS containing 10 µM EDTA. The first lavage was used for MCP-1 measurements. After BAL, lungs were perfused via the right ventricle with PBS, excised, minced and incubated in PBS containing collagenase (2.4 mg/ml; Roche Applied Science) and DNase (1 mg/ml; Roche Applied Science) for 30 minutes at 37°C. Single-cell suspensions were prepared by passage through cell strainers and lymphocytes were isolated using lympholyte-M (Cederlane) according to manufacturer’s instructions. BM cells were obtained by flushing the femurs and tibiae and single cell suspensions of individual spleens were prepared using cell strainers. Red blood cells were removed from the spleen and BM by ammonium chloride lysis.

### Antibodies and Flow Cytometry

Surface staining was performed for at least 20 minutes at 4°C in the presence of Fc-block (2.4G2). Fluorochrome-conjugated antibodies were purchased from eBioscience [CD49b (DX5), TCRβ (H57-597), NK1.1 (PK136), CD27 (LG.7F9), NKp46 (29A1.4), CD45.2 (104), CD45.1 (A20), KLRG1 (2F1), CD27 (M1/70)], and anti-CCR2 (475301) from R&D Systems. Samples were measured on a FACSCalibur or FACSCantoII (BD Biosciences) and analyzed with FlowJo software (Tree Star).

### Monocyte Chemotactic Protein-1 (MCP-1) ELISA

Levels of MCP-1 were determined in 1 ml of BALF (BAL fluid; supernatant of centrifuged BAL) by standard ELISA using clone 4E2 and biotinylated 2H5, both purchased from eBiosciences, as coating and detection antibody, respectively, according to manufacturer’s instructions. Recombinant MCP-1 (Biolegend) was used as a standard.

### Generation of Mixed BM Chimeras

Mixed BM chimeric mice were made as previously described [35]. In brief, CD4- and CD8- depleted BM cells of CD45.1 (*wild-type, wt*) and CCR2^−/−^ were 1-to-1 mixed and 10^7^ cells were transferred intravenously (*i.v.*) to lethally irradiated CD45.1.2. acceptor mice. Reconstituted mice were left for at least 42 days and then influenza virus infected as described.

## Results

### A Unique Population of CCR2^+^ NK Cells Resides in the BM, Spleen and Lungs of Naïve Mice

**Figure 1:**
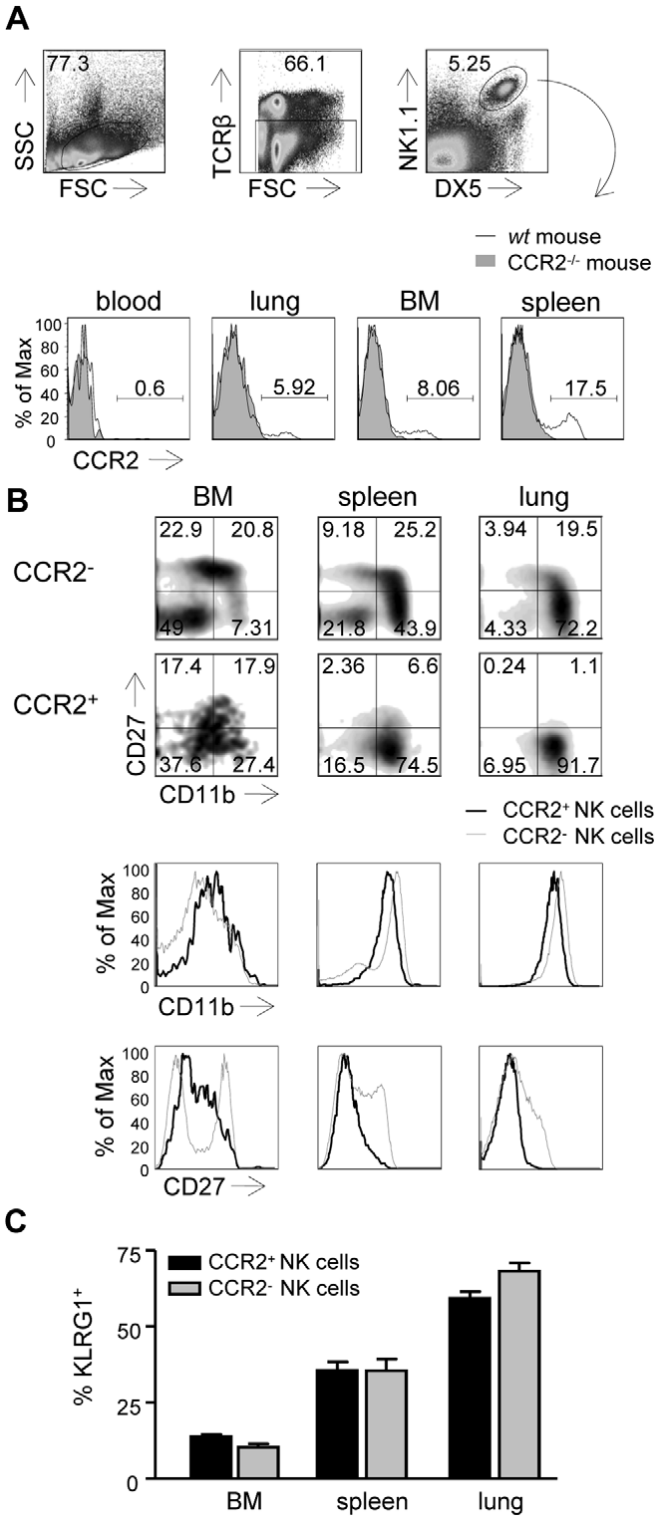
A subset of NK cells expresses the CCR2 receptor. (A) Gating strategy (upper panel) for NK cells (TCRβ^−^NK1.1^+^DX5^+^) and representative histograms (lower panel) showing CCR2 expression on NK cells recovered from the indicated organs (blood, lung, BM and spleen) of naïve C57BL/6 (*wt*) or CCR2^−/−^ mice. (B,C) Expression of CD27 and CD11b (C) or KLRG1 on electronically gated NK cells (TCRβ^−^NK1.1^+^) or CCR2^+^ NK cells. The depicted FACS plots are representatives of 9 mice that were analyzed in two independent experiments. Statistical analysis was performed using a Mann-Whitney U test (C) and KLRG-1-expression on CCR2^+^ or CCR2^−^ NK cells is not significantly different (Mann-Whitney U test, *, P<0.05).

In the current study we aimed to identify the role of CCR2 in NK cell migration during influenza virus infection. We first examined the expression of CCR2 on NK cells in several organs of naive mice using FACS analysis. Similar as in human blood [21], [36], NK cells in the blood of naïve mice did not express CCR2 (Figure 1A). In contrast, a clear population of NK cells in the spleen, BM and lung expressed CCR2, which was absent in CCR2^−/−^ mice (Figure 1A). To determine the developmental status of CCR2 expressing NK cells, we measured CD11b and CD27 expression by FACS analysis. NK cells that lack both CD11b and CD27 are considered to be immature and upon maturation, NK cells first upregulate CD27, then CD11b and the final maturation step involves CD27 downregulation [11], [12]. The CCR2^−^ NK cell population in the BM displayed an immature phenotype (CD11b^low^CD27^low^), CCR2^−^ NK cells in the spleen were mainly CD11b^high^CD27^high^ and CD11b^high^CD27^low^ indicating a more mature phenotype, and the lungs contained mostly mature CD11b^high^CD27^low^ CCR2^−^ NK cells (Figure 1B). In contrast, most of the CCR2^+^ NK cells in the lung and spleen lacked CD27 expression and although CD11b was present on most of these NK cells, the cell surface levels were lower than on CCR2^−^ NK cells (Figure 1B). In the BM, CCR2^+^ NK cells expressed intermediate levels of both CD27 and CD11b (Figure 1B) and thus, did not fall into any of the by CD27- and CD11b-expression defined maturation stages. We furthermore verified the expression of KLRG-1, which is acquired in a late stage of NK cell development [13]. A similar proportion of CCR2^−^ and CCR2^+^ NK cells expressed this marker in the lungs, spleen and BM (Figure 1C). Taken together, our results show that CCR2 expressing NK cells form a unique population in the BM, lungs and spleens of naive mice that, based on CD11b- and CD27-expression, distinguish themselves from NK cells that lack CCR2 expression.

### MCP-1 is Produced in the Airways of Influenza Virus Infected Mice

**Figure 2:**
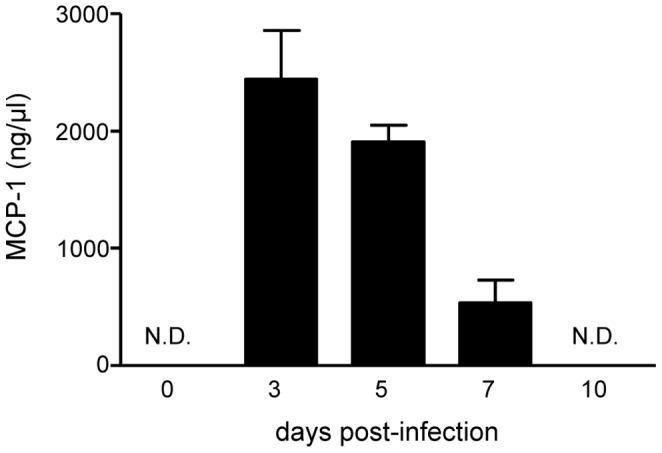
MCP-1 is expressed in the airways of influenza virus infected mice. (A) C57BL/6 mice were infected with HKx31 and the presence of MCP-1 in the BALF was determined by ELISA at the indicated days post-infection. Data shown are means+S.E.M. from one experiment with 5 mice per time point. Similar results were found in an independent experiment at day 2 and 5 post-infection.

To determine whether CCR2 may play a role in NK cell migration during infection, we next studied the expression levels of MCP-1, one of the CCR2 ligands, in the airways of influenza virus infected mice. We intranasally infected C57BL/6 mice with influenza virus HKx31 and at the indicated time points (Figure 2), levels of MCP-1 were determined in the BAL-fluid using ELISA. While absent from the airways of uninfected mice (d.0, Figure 2), influenza virus infection induced MCP-1 expression, which was clearly detectable in the BAL at day 3 of infection. Thereafter, MCP-1 levels declined gradually and were below the detection limit at day 10 post-infection (p.i.) (Figure 2). Thus, influenza virus infection induces MCP-1 production in the airways.

### CCR2-dependent NK Cell Migration During Influenza Virus Infection

**Figure 3:**
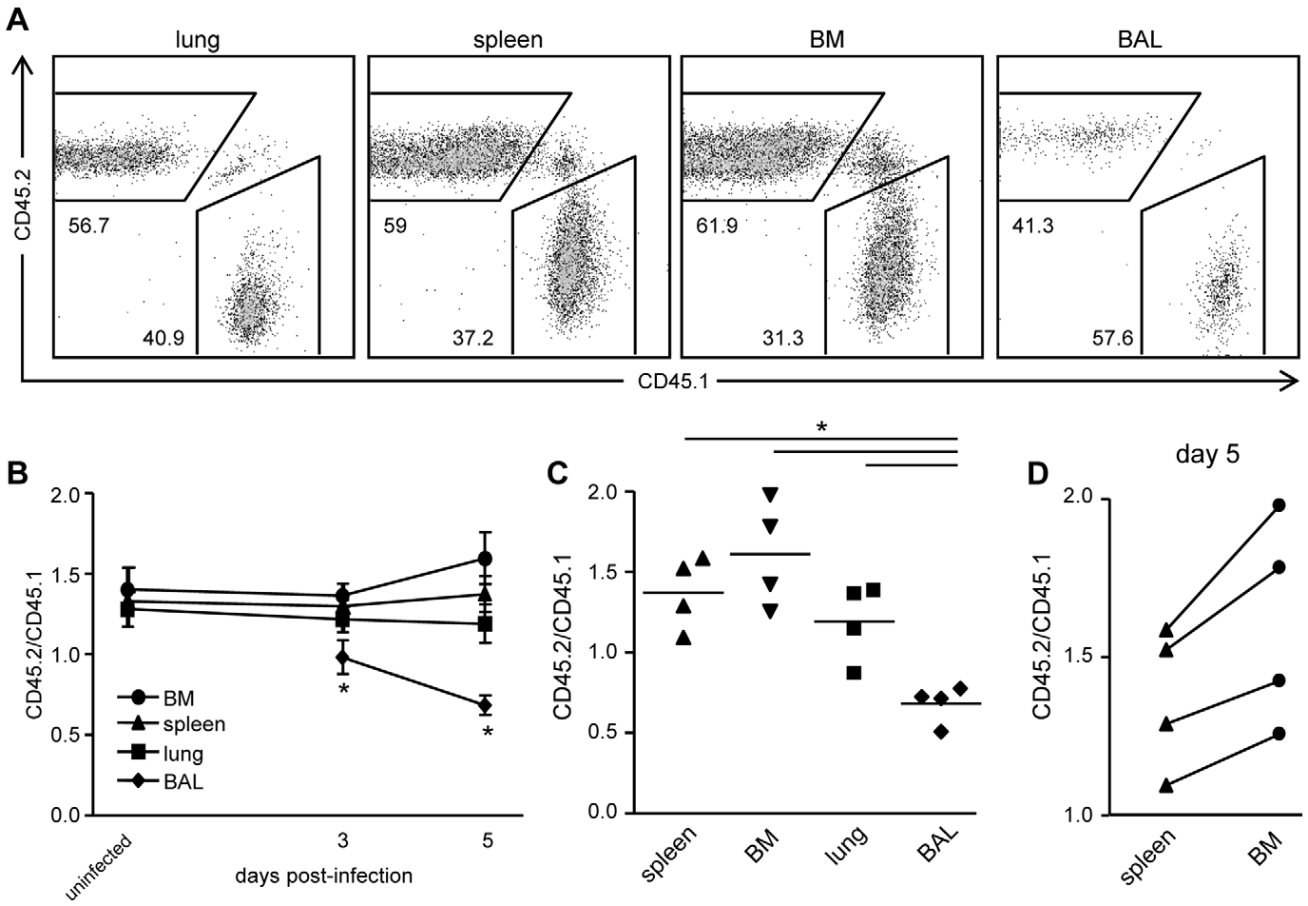
NK cells migrate CCR2-dependent to the BAL during influenza virus infection. Mixed BM chimeric mice were constructed by injecting a mix of BM from CCR2^−/−^ (CD45.2) and C57BL/6.SJL (CD45.1) into lethally irradiated CD45.1.2. recipients. After 6–8 weeks, mice were infected with influenza virus or left uninfected. (A) Representative FACS plots showing CD45.1 and CD45.2 staining of NK cells (TCRβ^−^NK1.1^+^) recovered from the indicated organs 5 days p.i. (B-D) Ratio of CD45.2 (CCR2^−/−^)/CD45.1 (*wt*) NK cells calculated by dividing absolute numbers of CD45.2^+^ NK cells by absolute numbers of CD45.1^+^ (*wt*) NK cells. Ratio of CD45.2/CD45.1 NK cells recovered from the indicated organs shown as average ± S.E.M. at the indicated days (B) and shown as individual mice at day 5 (C), and the ratio in the spleen and BM of individual mice are connected by a line at day 5 (D) after influenza virus infection. Representative results of two independent experiments are shown with 4–6 mice per group. Statistical analysis was performed using a Mann-Whitney U test. *, P<0.05.

To study whether NK cell migration occurs CCR2-dependent during influenza virus infection, and more specifically, whether this chemokine receptor mediates egress from the BM, mixed BM chimeric mice were constructed using BM from congenic *wt* (CD45.1) and CCR2^−/−^ (CD45.2) mice, which was transplanted into lethally irradiated congenic CD45.1.2. *wt* mice. After reconstitution for at least six weeks, similar ratios of NK cells derived from BM of *wt* (CD45.1) and CCR2^−/−^ (CD45.2) mice were found in the lungs, the spleen and the BM of reconstituted mice (Figure 3B). Upon infection with influenza virus, the relative proportions of CCR2^−/−^ (CD45.2) to *wt* (CD45.1) NK cells responding to infection in the spleen and lungs barely changed over the course of infection (day 3 and 5 p.i.) (Figure 3A, B)). In contrast, compared to *wt* (CD45.1) NK cells recovered from other organs, a significantly lower proportion of CCR2^−/−^ (CD45.2) NK cells was recovered from the BAL of influenza virus infected mice at both day 3 and 5 p.i and at day 5, an increase of CCR2^−/−^ NK cells in the BM was observed (Figure 3B, C). Although the differences were small, every individual mouse showed a relative increase in CCR2^−/−^ NK cell proportions, with an average increase of 15% (Figure 3D). Thus, in influenza virus infected CCR2^−/−^:*wt* mixed BM chimeric mice, relatively low proportions of CCR2^−/−^ compared to *wt* NK cells were recovered from the BAL.

## Discussion

In a recent report we have shown that influenza virus infection induced the accumulation of NK cells in the airways of mice [29] and we here show that a proportion of these NK cells required CCR2 for migration. To determine the role of CCR2 in migration, we constructed mixed *wt* (CD45.1) and CCR2^−/−^ (CD45.2) BM chimeric mice that allowed us to study the fate of CCR2^−/−^ compared to *wt* NK cells in several organs during influenza virus infection. Using these mixed BM chimeric mice, we consistently found that increased proportions of CCR2^−/−^ NK cells compared to *wt* NK cells accumulated in the BM during influenza virus infection (Figure 3), suggesting that this population required CCR2 for egress. As CCR2 is expressed by a small proportion of NK cells in the BM (Figure 1A), it is not unexpected that proportions of CCR2^−/−^ NK cells accumulating in the BM of influenza virus infected mice were relatively small. More dramatic differences, on the other hand, were found in the airways of these influenza virus infected mixed BM chimeric mice. Compared to other organs, we found a ∼50%-reduction in relative CCR2^−/−^ NK cell proportions in the BAL (Figure 3), indicating a clear requirement for CCR2 in NK cell migration during influenza virus infection. Interestingly, while relative proportions of CCR2^−/−^ NK cells were low in the BAL, the ratios of CCR2^−/−^ : *wt* NK cell in the lungs did not change over the course of infection. We currently cannot explain this discrepancy between the lung and BAL, however, also in a previous study where CD27/CD11b expression profiles were examined, we observed phenotypic differences between NK cells in the BAL and lungs [29].

Analysis of CCR2 expression on NK cells derived from uninfected *wt* C57BL/6 mice showed that NK cells isolated from the BM, spleen and lung express CCR2, while this chemokine receptor was absent on NK cells obtained from the blood (Figure 1A). Similarly, only a minor proportion of freshly isolated NK cells from the human blood express CCR2 and NK cell responsiveness towards MCP-1, one of the CCR2 ligands, is highly increased upon short-term activation [21], [36], [37]. During influenza virus infection, we find that less CCR2^−/−^ than *wt* NK cells accumulate in the airways, suggesting a role for CCR2 in NK cell migration. Although we cannot exclude that this is caused by factors other than migration, like e.g. preferential cell death of CCR2^−/−^ NK cells our data are in line with other studies that showed NK cells migration in response to CCR2 chemokines [21], [37], or demonstrated a role for CCR2 in NK cell migration to the site of infection. So was it shown that in neutrophil-depleted mice, MCP-1 was produced in the airways upon *Aspergillus fumigatus* infection, and this led to accumulation of cultivated adoptively transferred *wt* NK cells to the airways, which was significantly reduced when the transferred NK cells were CCR2-deficient [38]. MCP-1 neutralization and NK cell depletion led to higher mortality and fungal burdens in these mice [38]. Another report showed that in TAP-2 deficient patients that develop granulomatous lesions consisting of activated NK cells, CCR2 was highly expressed on cultured NK cells derived from these patients. Furthermore, MCP-1 levels in the BAL were unusually high, suggesting that MCP-1/CCR2 played a role in NK cell migration to the airways in these patients [39]. Also during MCMV infection, MCP-1- and CCR2-deficient mice showed reduced proportions of NK cells in the liver. It has, however, not been verified in this study whether MCP-1 and CCR2**-**deficiency had a direct or indirect effect on NK cell migration [28]. Taken together, in line with our study, these studies demonstrate that during inflammation, NK cells can be recruited in a CCR2-dependent manner to the site of infection.

Earlier studies have shown that during *L. monocytogenes* and influenza virus infection, TNF-α/inducible nitric oxide synthase (iNOS)-producing (Tip) DCs failed to accumulate at the site of infection in CCR2^−/−^ mice [33], [40]. Interestingly, CCR2 was not involved in moving from the bloodstream to the infected tissues of *L. monocytogenes*-infected mice, but mediated egress of Tip-DC precursors from the BM to the blood [31]. We here aimed to study whether CCR2 mediates NK cell egress from the BM during influenza virus infection. We found a dramatically lower ratio of CCR2^−/−^ : *wt* NK cells in the airways of influenza virus infected CCR2^−/−^:*wt* mixed BM chimeric mice. However, at the same time, only a small accumulation of CCR2^−/−^ NK cells in the BM was observed, suggesting that only a small proportion of NK cells required CCR2 to egress from the BM. To further unravel the role of CCR2 in NK cell migration, we adoptively transferred a mix of CCR2^−/−^ and *wt* NK cells to influenza virus infected mice via the intravenous route. These NK cells were isolated from the spleens or BM of donor mice, and the injection into the bloodstream allowed us to study their capacity to migrate to the airways, without having to egress from the BM. We sacrificed the donor mice at several days post-infection, but found that adoptively transferred NK cells migrated preferably to the organs from which they originated (e.g. spleen or BM). We could recover only a small number of adoptively transferred NK cells from the BAL, making it impossible to draw reliable conclusions on the role of CCR2 in NK cell migration from the blood into the lungs (results not shown). We therefore cannot decipher whether the decreased proportions of CCR2^−/−^ NK cells in the BAL are the result of diminished egress from the BM, diminished migration to the airways, or a combination of the two.

In conclusion, we here show that a proportion of NK cells express the chemokine receptor CCR2 and migrate in a CCR2-dependent fashion to the airways during influenza virus infection. CCR2^+^ NK cells are phenotypically different from CCR2^−/−^ NK cells and therefore might fulfill a unique role in the immune system.
